# Supplementary material for: A novel genus and cryptic species harboured within the monotypic freshwater crayfish genus Tenuibranchiurus Riek, 1951 (Decapoda: Parastacidae)
Source: PeerJ. 2017 May 24;5:e3310. doi: 10.7717/peerj.3310 (PMC5445942; doi:10.7717/peerj.3310)
Supplement: Table S1 — All samples used in this study to infer the individual gene trees. * indicates samples obtained from GenBank, QB indicates samples obtained from Q. Burnham unpublished data, and all others are from this study. Where samples have more than one ID name, the first is the identification used in this study, and the second is the identifier from GenBank. [file peerj-05-3310-s001.docx]

Table S1. All samples used in this study to infer the individual gene trees. * indicates samples obtained from GenBank, QB indicates samples obtained from Q. Burnham unpublished data, and all others are from this study. Where samples have more than one ID name, the first is the identification used in this study, and the second is the identifier from GenBank.

| Genus | Species | Sample ID | GenBank Accession Number | | | | |
| --- | --- | --- | --- | --- | --- | --- | --- |
|  |  |  | COI | 16S | GAPDH | H3 | AK |
| *Tenuibranchiurus* | *glypticus* | TL1/BEL1.2 | - | EU977397* | EU977450* | - | - |
|  |  | TL2_1 | - | KX669691 | - | - | - |
|  |  | TL2_2 | - | - | - | KX670023 | - |
|  |  | TL2_3 | - | - | - | - | KX669746 |
|  |  | TL2/BEL2.2 | - | EU977398* | EU977451* | - | - |
|  |  | TL3_1 | KX753349 | KX669692 | - | - | - |
|  |  | TL3 | - | AF135998* | - | - | - |
|  |  | BRB1_1/BRB1.1 | - | - | EU977452* | - | - |
|  |  | BRB2_1 | KX669801 | - | - | - | - |
|  |  | BRB2_3 | KX669802 | - | - | - | - |
|  |  | BRB2_6 | KX669803 | - | - | - | - |
|  |  | BRB2_R1 | KX669804 | - | - | - | - |
|  |  | BRB_1 | KX669805 | KX669693 | - | KX670024 | KX669747 |
|  |  | BRB_2 | KX669806 | KX669694 | - | KX670025 | KX669748 |
|  |  | BRB_3 | KX669807 | KX669695 | - | KX670026 | KX669749 |
|  |  | BRB_4 | KX669808 | KX669696 | - | KX670027 | KX669750 |
|  |  | BRB_5 | KX669809 | KX669697 | - | KX670028 | KX669751 |
|  |  | BRB_6 | KX669810 | KX669698 | - | KX670029 | KX669752 |
|  |  | Moo | - | EF493133* | - | - | - |
|  |  | BER_1 | KX669811 | - | KX669932 | - | - |
|  |  | BER_2 | KX669812 | KX669699 | KX669933 | KX670030 | KX669753 |
|  |  | BER_5 | KX669813 | - | KX669934 | - | - |
|  |  | TSFSC_1 | KX669814 | - | KX669935 | - | - |
|  |  | TSFSA_2 | KX669815 | - | KX669936 | - | - |
|  |  | TSFSA_3 | KX669816 | - | KX669937 | - | - |
|  |  | TSFSE_5 | KX669817 | KX669700 | KX669938 | KX670031 | KX669754 |
|  |  | TSFSA_6 | KX669818 | - | KX669939 | - | - |
|  |  | TSFSF_7 | KX669819 | - | KX669940 | - | - |
|  |  | TSFSG_9 | KX669820 | - | KX669941 | - | - |
|  |  | TSFSH_10 | KX669821 | - | KX669942 | - | - |
|  |  | TSFSC_11 | KX669822 | - | KX669943 | - | - |
|  |  | TSFSC_12 | KX669823 | - | KX669944 | - | - |
|  |  | TSFSC_13 | KX669824 | - | - | KX670032 | KX669755 |
|  |  | TSFSC_14 | KX669825 | - | KX669945 | - | - |
|  |  | TSFSC_15 | KX669826 | - | KX669946 | - | - |
|  |  | TSFSC_16 | KX669827 | - | - | - | - |
|  |  | TSFSE_18 | KX669828 | - | KX669947 | - | - |
|  |  | TSFSC_19 | KX669829 | KX669701 | KX669948 | KX670033 | KX669756 |
|  |  | TSFSE_20 | KX669830 | KX669702 | KX669949 | KX670034 | KX669757 |
|  |  | TSFSG_21 | KX669831 | - | KX669950 | - | - |
|  |  | TSFSC_23 | KX669832 | - | KX669951 | - | - |
|  |  | TSFSF_24 | KX669833 | - | KX669952 | - | - |
|  |  | TSFSF_25 | KX669834 | KX669703 | KX669953 | KX670035 | KX669758 |
|  |  | TSFSG_26 | KX669835 | - | KX669954 | - | - |
|  |  | TSFSC_27 | KX669836 | - | KX669955 | - | - |
|  |  | TSFSG_28 | KX669837 | - | KX669956 | - | - |
|  |  | TSFSC_29 | KX669838 | - | KX669957 | - | - |
|  |  | TSFSE_30 | KX669839 | - | KX669958 | - | - |
| *Tenuibranchiurus* | sp. nov. 1 | KNP | - | EF493131* | - | - | - |
|  |  | MAR_1 | KX669840 | KX669704 | KX669959 | KX670036 | - |
|  |  | MAR_2 | - | - | KX669960 | - | - |
|  |  | MAR_3 | KX669841 | KX669705 | KX669961 | KX670037 | KX669759 |
|  |  | MAR_4 | KX669842 | - | - | - | - |
|  |  | MAR_5 | KX669843 | - | - | - | - |
|  |  | MAR_6 | KX669844 | - | KX669962 | - | - |
|  |  | MAR_7 | - | - | KX669963 | - | - |
|  |  | MAR_8 | KX669845 | - | KX669964 | - | - |
|  |  | MAR_9 | KX669846 | KX669706 | KX669965 | KX670038 | KX669760 |
|  |  | MAR_10 | KX669847 | - | KX669966 | KX670039 | KX669761 |
|  |  | MAR_11 | KX669848 | KX669707 | - | KX670040 | - |
|  |  | MAR_12 | KX669849 | - | KX669967 | - | - |
|  |  | TSFN_12 | KX669850 | KX669708 | - | - | - |
|  |  | TSFN_15 | KX669851 | KX669709 | - | - | - |
|  |  | TSFSA_4 | KX669852 | KX669710 | KX669968 | KX670041 | KX669762 |
|  |  | TSFSC_8 | KX669853 | - | KX669969 | - | - |
|  |  | TSFSC_17 | KX669854 | KX669711 | KX669970 | KX670042 | KX669763 |
|  |  | TSFSC_22 | KX669855 | KX669712 | KX669971 | KX670043 | KX669764 |
| *Tenuibranchiurus* | sp. nov. 2 | BER_3 | KX669856 | - | KX669972 | - | - |
|  |  | BER_4 | KX669857 | KX669713 | - | KX670044 | KX669765 |
|  |  | BER_6 | KX669858 | - | - | - | - |
|  |  | BER_7 | KX669859 | - | KX669973 | - | - |
| *Tenuibranchiurus* | sp. nov. 3 | HB_1 | KX669860 | KX669714 | - | KX670045 | KX669766 |
|  |  | HB_2 | - | - | - | KX670046 | KX669767 |
|  |  | HB_4 | - | KX669715 | - | KX670047 | KX669768 |
|  |  | HB_5 | - | KX669716 | - | KX670048 | KX669769 |
|  |  | HB_S2 | - | KX669717 | - | - | - |
| *Tenuibranchiurus* | sp. nov. 4 | TEW_1 | KX669861 | KX669718 | KX669974 | KX670049 | KX669770 |
|  |  | TEW_2 | KX669862 | KX669719 | KX669975 | KX670050 | KX669771 |
|  |  | TEW_3 | KX669863 | - | - | - | - |
|  |  | TEW_4 | KX669864 | - | KX669976 | - | KX669772 |
|  |  | TEW_5 | KX669865 | - | KX669977 | KX670051 | KX669773 |
|  |  | TEW_6 | KX669866 | - | KX669978 | - | - |
|  |  | TEW_7 | KX669867 | - | KX669979 | KX670052 | - |
|  |  | TEW_T1/TEW1.1 | - | EU977400* | EU977453* | - | - |
|  |  | LW_1 | KX669868 | KX669720 | KX669980 | KX670053 | KX669774 |
|  |  | LW_2 | KX669869 | KX669721 | KX669981 | KX670054 | KX669775 |
|  |  | LW_3 | KX669870 | - | KX669982 | KX670055 | KX669776 |
|  |  | LW_4 | KX669871 | - | KX669983 | - | - |
|  |  | LW_5 | KX669872 | KX669722 | KX669984 | KX670056 | KX669777 |
|  |  | LW_6 | KX669873 | - | KX669985 | - | - |
|  |  | LW_7 | KX669874 | KX669723 | KX669986 | KX670057 | - |
|  |  | Eu | - | EF493132* | - | - | - |
|  | sp. nov. 5 | GC1_1 | KX669875 | KX669724 | KX669987 | KX670058 | KX669778 |
|  |  | GC1_2 | KX669876 | - | KX669988 | KX670059 | - |
|  |  | GC1_3 | KX669877 | - | KX669989 | KX670060 | KX669779 |
|  |  | GC1_4 | KX669878 | KX669725 | - | KX670061 | - |
|  |  | GC1_5 | KX669879 | KX669726 | KX669990 | KX670062 | KX669780 |
|  |  | GC1_6 | KX669880 | - | KX669991 | - | - |
|  |  | GC1_8 | KX669881 | - | - | - | - |
|  |  | GC1_9 | KX669882 | - | - | - | - |
|  |  | GC2_1 | KX669883 | - | - | KX670063 | KX669781 |
|  |  | GC2_2 | KX669884 | - | KX669992 | - | - |
|  |  | GC2_3 | KX669885 | KX669727 | KX669993 | KX670064 | KX669782 |
|  |  | GC2_4 | KX669886 | KX669728 | KX669994 | KX670065 | KX669783 |
|  |  | GC2_5 | KX669887 | KX669729 | KX669995 | KX670066 | - |
|  |  | GC2_6 | KX669888 | - | KX669996 | - | - |
|  |  | GC2_7 | KX669889 | - | KX669997 | - | - |
| *Gen. nov.* | sp. nov. 1 | LH_1 | KX669890 | KX669730 | - | KX670067 | - |
|  |  | LH_2 | KX669891 | - | - | - | - |
|  |  | LH_3 | KX669892 | - | KX669998 | - | - |
|  |  | LH_4 | KX669893 | - | KX669999 | - | - |
|  |  | LH_5 | KX669894 | - | KX670000 | - | - |
|  |  | LH_6 | KX669895 | KX669731 | KX670001 | KX670068 | KX669784 |
|  |  | LH_7 | KX669896 | - | - | - | - |
|  |  | LH_8 | KX669897 | KX669732 | KX670002 | KX670069 | KX669785 |
|  |  | LH_9 | KX669898 | - | KX670003 | - | - |
|  |  | LH_10 | KX669899 | - | KX670004 | - | - |
|  |  | LH_11 | KX669900 | - | KX670005 | - | - |
|  |  | LH_13 | KX669901 | - | KX670006 | - | - |
|  |  | LH_14 | KX669902 | - | KX670007 | - | - |
|  |  | LH_15 | - | KX669733 | - | KX670070 | KX669786 |
| *Gen. nov.* | sp. nov. 2 | BNP1_1 | KX669903 | KX669734 | KX670008 | KX670071 | KX669787 |
|  |  | BNP1_2 | KX669904 | - | KX670009 | - | - |
|  |  | BNP1_3 | KX669905 | - | KX670010 | - | - |
|  |  | BNP1_4 | KX669906 | - | KX670011 | - | - |
|  |  | BNP1_5 | KX669907 | - | - | - | - |
|  |  | BNP1_6 | KX669908 | - | KX670012 | - | - |
|  |  | BNP1_7 | KX669909 | - | KX670013 | - | - |
|  |  | BNP1_8 | KX669910 | KX669735 | KX670014 | KX670072 | - |
|  |  | BNP1_9 | KX669911 | - | KX670015 | - | - |
|  |  | BNP1_10 | KX669912 | KX669736 | - | KX670073 | KX669788 |
|  |  | BNP1_11 | KX669913 | - | KX670016 | - | - |
|  |  | BNP1_12 | KX669914 | - | - | - | - |
|  |  | BNP1_13 | KX669915 | KX669737 | - | KX670074 | - |
|  |  | BNP2_1 | KX669925 | - | KX670017 | - | - |
|  |  | BNP2_2 | KX669926 | KX669738 | KX670018 | KX670075 | - |
|  |  | LakeH_1 | KX669916 | KX669739 | KX670019 | KX670076 | KX669789 |
|  |  | LakeH_2 | KX669917 | - | KX670020 | - | - |
|  |  | LakeH_3 | KX669918 | - | KX670021 | KX670077 | KX669790 |
|  |  | LakeH_4 | KX669919 | - | - | - | - |
|  |  | LakeH_5 | KX669920 | - | - | - | - |
|  |  | LakeH_6 | KX669921 | - | KX670022 | KX670078 | - |
|  |  | LakeH_7 | KX669922 | - | - | - | - |
|  |  | LakeH_18 | KX669923 | KX669740 | - | KX670079 | KX669791 |
|  |  | LakeH_19 | KX669924 | KX669741 | - | KX670080 | KX669792 |
| *Cherax* | *glaber* | Cherax_glaber | - | - | - | DQ079670* | - |
| *Cherax* | *quinquecarinatus* | Cherax_quinquecarinatus | HM641111* | - | - | - | - |
| *Cherax* | *robustus* | Cherax_robustus | - | EU977343* | EU977412* | - | - |
| *Engaeus* | *fossor* | Engaeus_fossor | EU921144* | - | - | - | - |
| *Engaeus* | *sericatus* | Engaeus_sericatus | FJ965960* | - | - | - | - |
| *Engaeus* | *lyelli* | 892 | - | QB | - | KX670081 | - |
| *Engaeus* | *laevis* | 928 | - | QB | QB | KX670082 | KX669793 |
| *Engaeus* | *mallacoota* | 1014 | - | - | QB | KX670083 | - |
| *Engaewa* | *pseudoreducta* | 104 | QB | JQ613110* | QB | KX670084 | - |
|  |  | 125 | QB | JQ613118* | QB | KX670085 | KX669794 |
|  | *reducta* | 124 | QB | JQ613117* | QB | KX670086 | KX669795 |
| *Geocharax* | *falcata* | 3632 | AF493632* | - | - | - | - |
|  |  | 7437/SWC1.1 |  | EF493144* | EU977437* | - | - |
|  | *gracilis* | 330 | QB | QB | QB | KX670087 | KX669796 |
|  |  | 1145 | EU921145* | - | - | - | - |
|  |  | 3150 | - | EF493150* | - | - | - |
|  |  | 7439/MSQ1.3 | - | EF493095* | EU977439* | - | - |
| *Gramastacus* | *insolitus* | 887 | QB | QB | - | KX670088 | KX669797 |
|  |  | 1062 | - | - | - | EU921062* |  |
|  |  | 3043 | - | EF493043* | - | - | - |
|  |  | 7445/3BX1.1 | - | EF493040* | EU977445* | - | - |
|  |  | 7446/DWY3.1 | - | EF493066* | EU977446* | - | - |
|  | *lacus* | M1 | KX669927 | KX669742 | - | KX670089 | KX669798 |
|  |  | M2 | KX669928 | KX669743 | - | KX670090 | - |
|  |  | M3 | KX669929 | KX669744 | - | KX670091 | KX669799 |
|  |  | M4 | KX669930 | KX669745 | - | KX670092 | - |
|  |  | M5 | KX669931 | - | - | KX670093 | KX669800 |
|  |  | 7447/MYL1.1 | - | EF493118* | EU977447* | - | - |
|  |  | 7448/MYL1.3 | - | EF493120* | EU977448* | - | - |
