# Supplementary material for: A novel genus and cryptic species harboured within the monotypic freshwater crayfish genus Tenuibranchiurus Riek, 1951 (Decapoda: Parastacidae)
Source: PeerJ. 2017 May 24;5:e3310. doi: 10.7717/peerj.3310 (PMC5445942; doi:10.7717/peerj.3310)
Supplement: Table S2 — Process of assigning genetic groups/populations to potential lineages within Queensland. [file peerj-05-3310-s002.docx]

Table S2. Process of assigning genetic groups/populations to potential lineages within Queensland.

| Number of potential lineages | Explanation | Graphical representation | Populations included |
| --- | --- | --- | --- |
| 2 | Major division within Qld | 1  2  5  2  3  4  1  1  2  3  4  5  6  1  2  3  4  5  6  7  8  1  3  2  1  2  3  4  5  6  7  8  9  10  1  2  3  4  5  6  7  8  9  10  11  1  2  3  4  5  6  7  8  9  10  11  12  13 | 1 = MAR, TSFN, TSFS, TL, BRB, BER  2 = HB, BER, TEW, LW, GC |
| 3 | Major division within top portion of tree |  | 1 = MAR, TSFN, TSFS  2 = TSFS, TL, BRB, BER  3 = HB, BER, TEW, LW, GC |
| 5 | Major divisions within bottom portion of tree |  | 1 = MAR, TSFN, TSFS, TL, BRB, BER  2 = BER  3 = HB  4 = TEW, LW  5 = GC |
| 6 | Major divisions within both top and bottom portion of tree |  | 1 = MAR, TSFN, TSFS  2 = TSFS, TL, BRB, BER  3 = BER  4 = HB  5 = TEW, LW  6 = GC |
| 8 | Minor divisions within both top and bottom portion of tree |  | 1 = MAR  2 = TSFN, TSFS  3 = TSFS  4 = TL, BRB, BER  5 = BER  6 = HB  7 = TEW, LW  8 = GC |
| 10 | All geographic localities separated within bottom portion of tree |  | 1 = MAR  2 = TSFN, TSFS  3 = TSFS  4 = TL, BRB, BER  5 = BER  6 = HB  7 = TEW  8 = LW  9 = GC1  10 = GC2 |
| 11 | All geographic localities separated within top portion of tree |  | 1 = MAR  2 = TSFN  3 = TSFS  4 = TSFS  5 = TL  6 = BRB  7 = BER  8 = BER  9 = HB  10 = TEW, LW  11 = GC |
| 13 | All geographic localities separated within entire tree |  | 1 = MAR  2 = TSFN  3 = TSFS  4 = TSFS  5 = TL  6 = BRB  7 = BER  8 = BER  9 = HB  10 = TEW  11 = LW  12 = GC1  13= GC2 |
